# Supplementary material for: Structure of the cytoplasmic ring of the Xenopus laevis nuclear pore complex by cryo-electron microscopy single particle analysis
Source: Cell Res. 2020 May 6;30(6):520–31. doi: 10.1038/s41422-020-0319-4 (PMC7264146; doi:10.1038/s41422-020-0319-4)
Supplement: Supplementary file 1 — Supplementary Figure S1 [file 41422_2020_319_MOESM1_ESM.pdf]

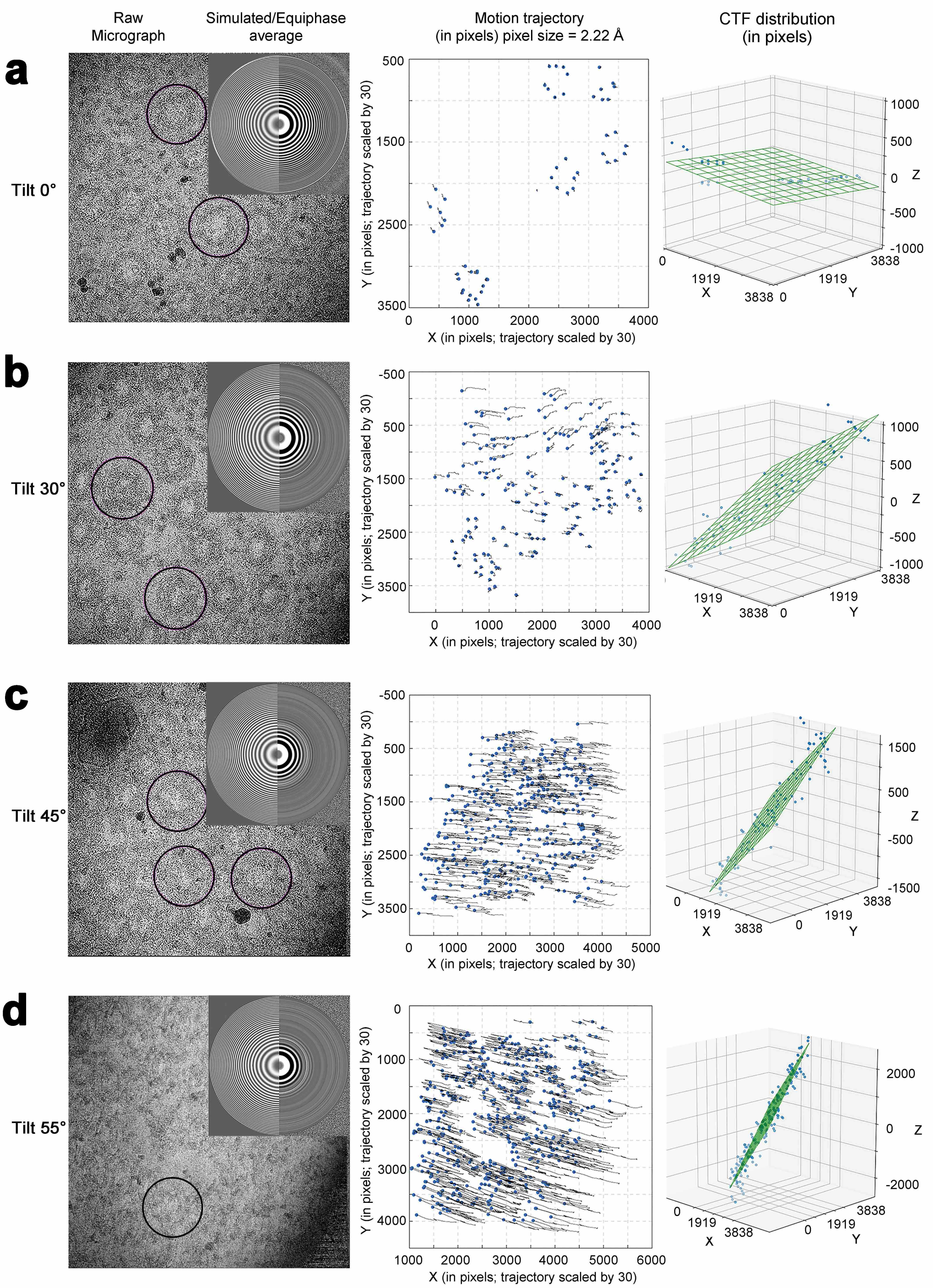

Supplementary information, Fig. S1 | Quality analysis of the cryo-EM data for the NPC from *X. laevis* oocytes. **a**, Representative data from the tilt-0° series. A representative raw micrograph, the motion trajectory summary, and the CTF

distribution are shown in the left, middle, and right panels, respectively. The results of CTF estimation using Gctf[1] are shown in the upper right corner of the raw micrograph (left panel). The white circles represent the estimated resolution limit. **b**, Representative data from the tilt-30° series. **c**, Representative data from the tilt-45° series. **d**, Representative data from the tilt-55° series. As the tilting angle increases, the quality of the micrograph gradually decreases. The decrease of quality mainly originates from two aspects. First, particle motion increases as the tilting angle increases (middle panels), because tilting the sample plane projects the doming effect of the Z-direction onto the X-Y plane. Second, sample thickness is inversely proportional to the cosine of the tilting angle, resulting in an increased noise level in the micrograph. Two deliberate measures were taken to alleviate the problem of decreased quality at high tilting angles. First, the total electron dose followed an inverse-cosine scheme to increase contrast at high tilting angles. Second, more micrographs were collected at higher tilting angles. The third but unintentional measure is that a micrograph at a higher tilting angle includes more particles because the view field of on the sample plane is inversely proportional to the cosine of the tilting angle.

Reference

1 Zhang, K. Gctf: Real-time CTF determination and correction. *J Struct Biol* **193**, 1-12, doi:10.1016/j.jsb.2015.11.003 (2016).
