# Supplementary material for: Structure of the cytoplasmic ring of the Xenopus laevis nuclear pore complex by cryo-electron microscopy single particle analysis
Source: Cell Res. 2020 May 6;30(6):520–31. doi: 10.1038/s41422-020-0319-4 (PMC7264146; doi:10.1038/s41422-020-0319-4)
Supplement: Supplementary file 2 — Supplementary Figure S2 [file 41422_2020_319_MOESM2_ESM.pdf]

Supplementary information, Fig. S2

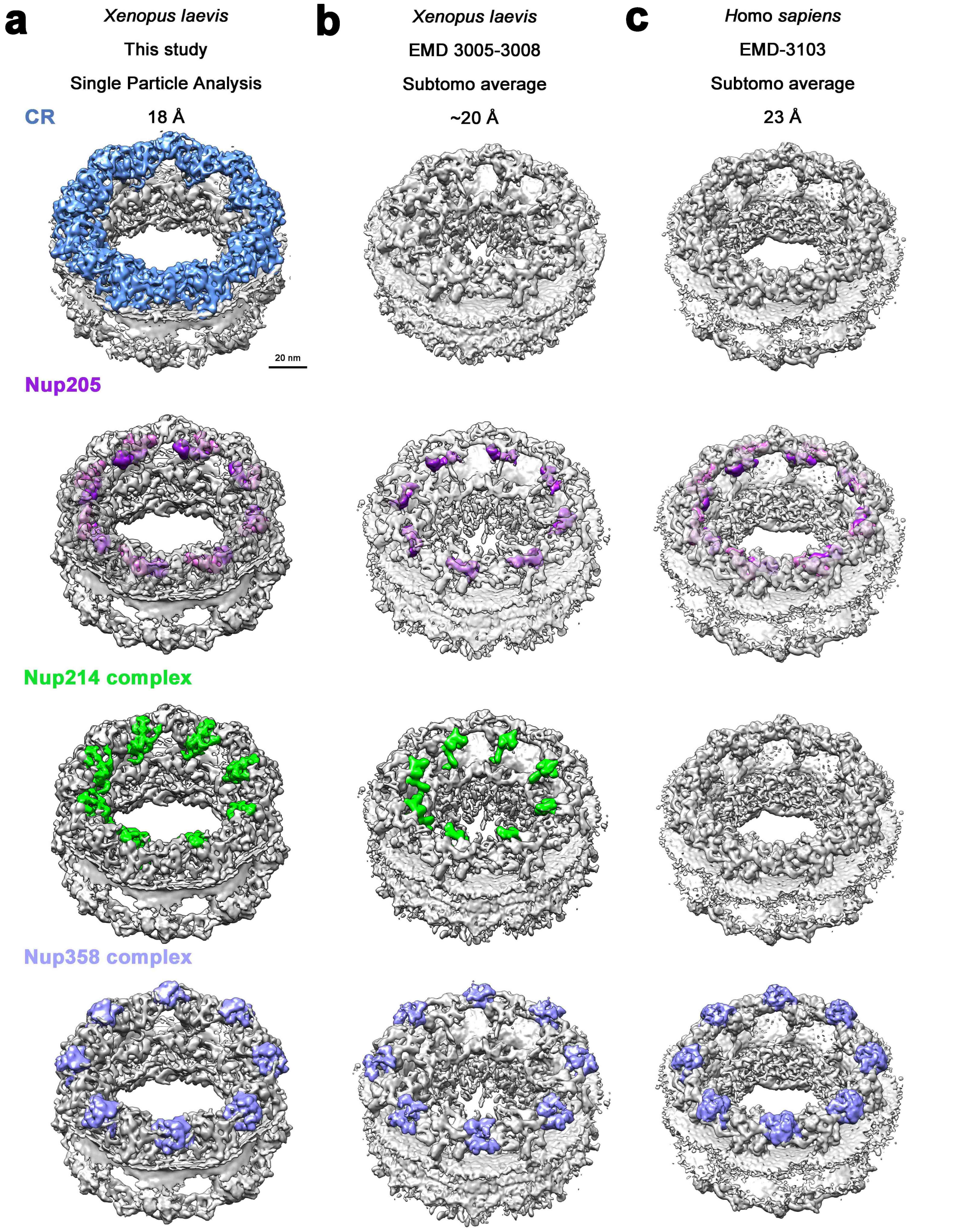

Supplementary information, Fig. S2 | Structural comparison of the CR from representative studies. **a**, Our preliminary 18.3-Å reconstruction of the CR using single particle analysis. From top to bottom, the four panels display features of the overall CR (colored marine), Nup205 or Nup188 (magenta), the Nup214 complex (green) and the Nup358 complex (purple). **b**, Cryo-ET structure of the *X. laevis* NPC

using sub-tomogram averaging (STA) (EMD 3005-3008)[1]. **c**, Cryo-ET structure of the *Homo sapiens* (*H. sapiens*) NPC using STA (EMD-3103)[2]. Although the EM map of human NPC was used as the initial reference, our reconstruction more closely resembles the cryo-ET reconstruction of the *X. laevis* NPC in terms of both overall shape and fine features. In particular, the Nup214 complex resembles the cryo-ET *X. laevis* NPC more than the cryo-ET human NPC. Therefore, our reconstruction was not biased by the initial reference used. Subsequent analysis at the CR subunit level shows a plethora of structural details that were previously unrecognized in low-resolution reconstructions.

References

1 Eibauer, M. *et al*. Structure and gating of the nuclear pore complex. *Nat Commun* **6**, 7532, doi:10.1038/ncomms8532 (2015).

2 von Appen, A. *et al*. In situ structural analysis of the human nuclear pore complex. *Nature* **526**, 140-143, doi:10.1038/nature15381 (2015).
