# Supplementary material for: Structure of the cytoplasmic ring of the Xenopus laevis nuclear pore complex by cryo-electron microscopy single particle analysis
Source: Cell Res. 2020 May 6;30(6):520–31. doi: 10.1038/s41422-020-0319-4 (PMC7264146; doi:10.1038/s41422-020-0319-4)
Supplement: Supplementary file 3 — Supplementary Figure S3 [file 41422_2020_319_MOESM3_ESM.pdf]

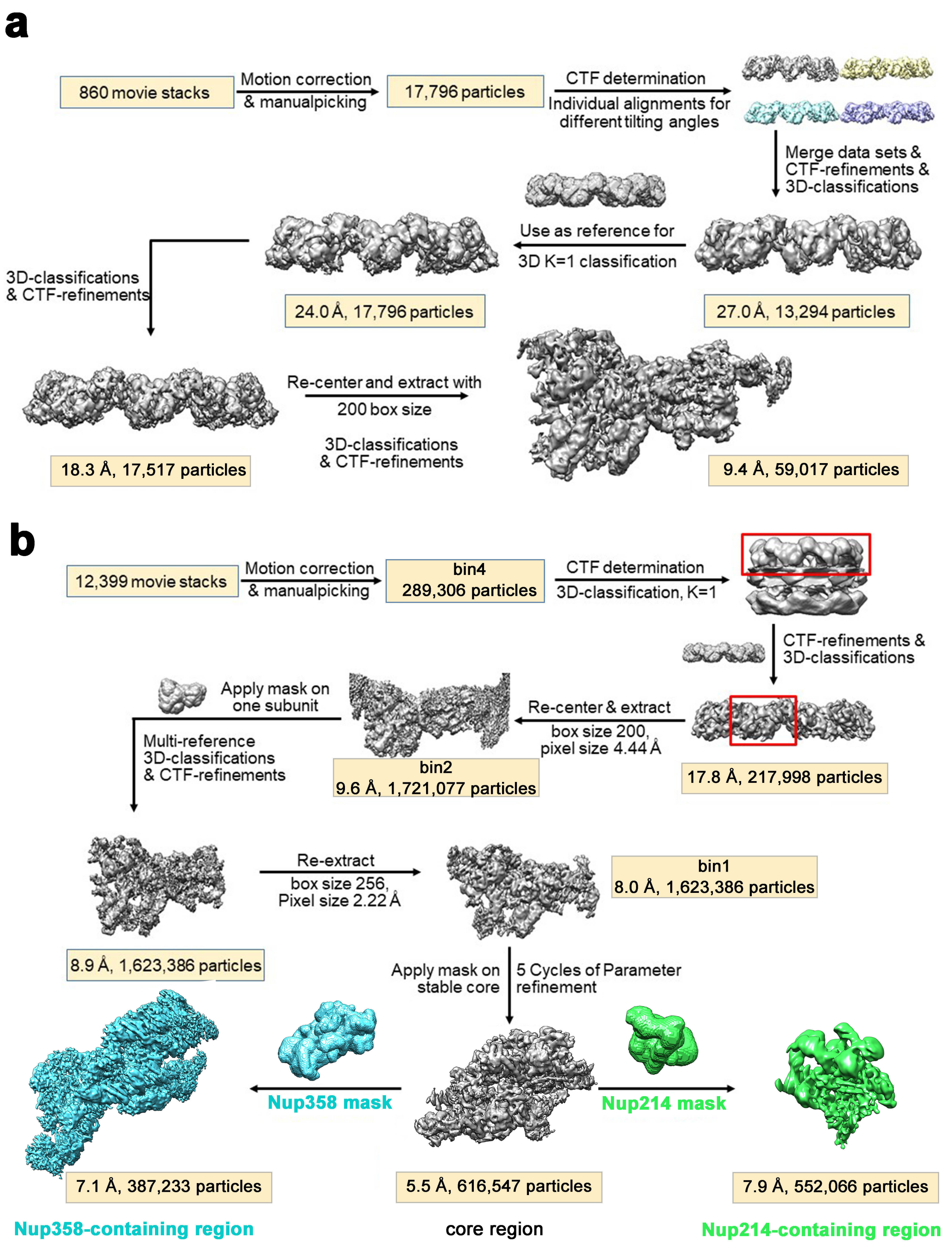

Supplementary information, Fig. S3 | Cryo-EM data processing. **a**, A flowchart diagram of preliminary cryo-EM data processing. Only 17,796 particles from 860 movie stacks were used. Details of the data processing are described in the MATERIALS AND METHODS. **b**, A flowchart diagram of the complete cryo-EM data processing. The CR subunit is divided into three overlapping regions: the Core

region, the Nup358-containing region, and the Nup214-containing region. The final reconstructions of the Core region, the Nup358-containing region, and the Nup214-containing region display average resolutions of 5.5 Å, 7.1 Å and 7.9 Å, respectively.
