# Supplementary material for: Structure of the cytoplasmic ring of the Xenopus laevis nuclear pore complex by cryo-electron microscopy single particle analysis
Source: Cell Res. 2020 May 6;30(6):520–31. doi: 10.1038/s41422-020-0319-4 (PMC7264146; doi:10.1038/s41422-020-0319-4)
Supplement: Supplementary file 4 — Supplementary Figure S4 [file 41422_2020_319_MOESM4_ESM.pdf]

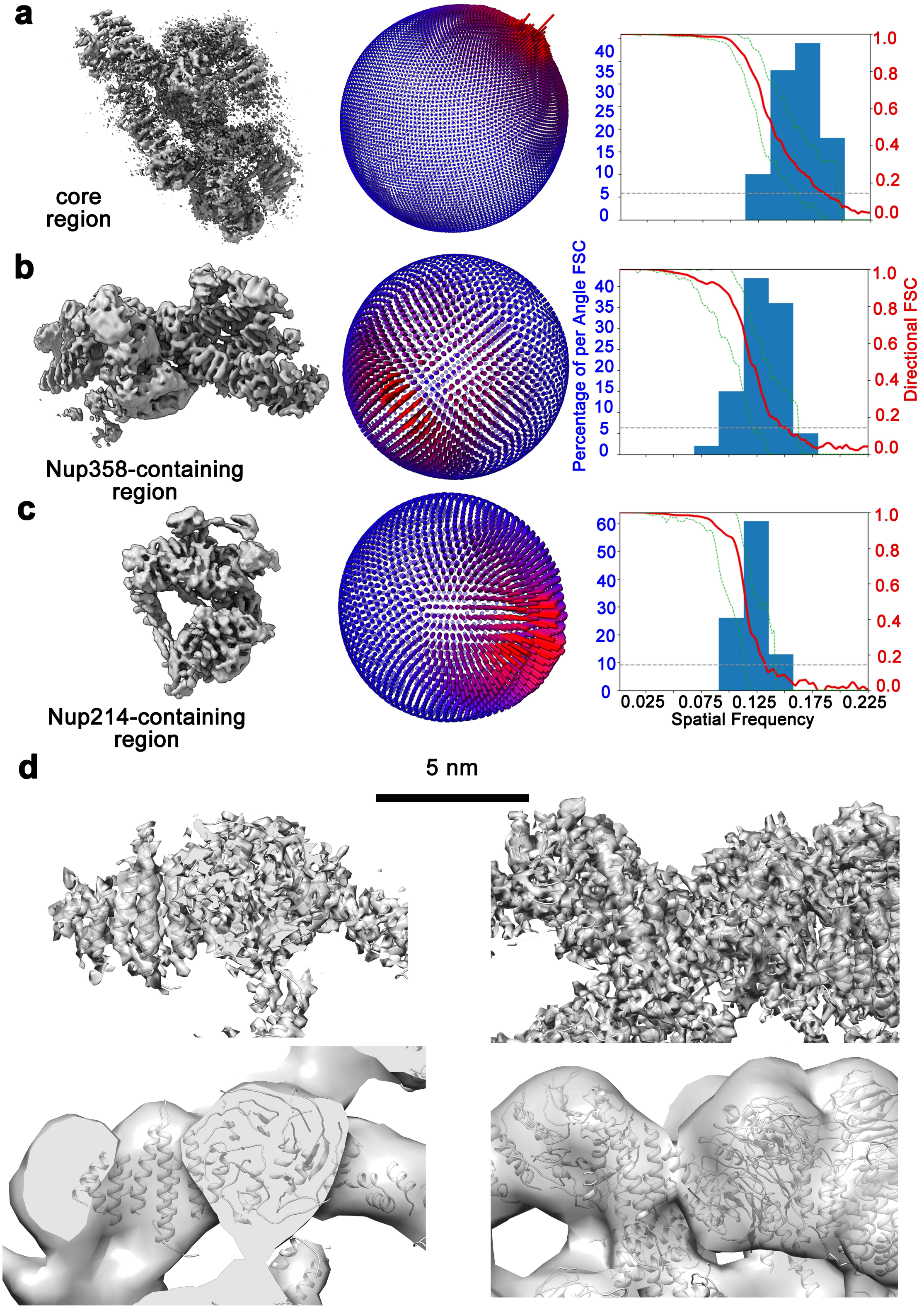

Supplementary information, Fig. S4 | The quality of cryo-EM reconstruction for the CR subunit. **a**, The quality of cryo-EM reconstruction for the Core region. The EM density, angular distribution of the particles used for the final reconstruction, and

directional FSC of the Core region are shown in the left, middle, and right panels, respectively. In the angular distribution plot, each cylinder represents one view and the height of the cylinder is proportional to the number of particles for that view. **b**, The quality of cryo-EM reconstruction for the Nup358-containing region. **c**, The quality of cryo-EM reconstruction for the Nup214-containing region. All directional FSC curves were prepared using the following website: https://3dfsc.salk.edu[1]. **d**, Comparison of our cryo-EM reconstruction of the *X. laevis* CR with that of the *H. sapiens* CR[2]. The same regions of the reconstruction are shown for the cryo-EM reconstruction of the *X. laevis* CR (upper panels) and the cryo-ET reconstruction of the *H. sapiens* CR[2] (lower panels).

References

1 Tan, Y. Z. *et al*. Addressing preferred specimen orientation in single-particle cryo-EM through tilting. *Nat Methods* **14**, 793-796, doi:10.1038/nmeth.4347 (2017).

2 von Appen, A. *et al*. In situ structural analysis of the human nuclear pore complex. *Nature* **526**, 140-143, doi:10.1038/nature15381 (2015).
