# Supplementary material for: Structure of the cytoplasmic ring of the Xenopus laevis nuclear pore complex by cryo-electron microscopy single particle analysis
Source: Cell Res. 2020 May 6;30(6):520–31. doi: 10.1038/s41422-020-0319-4 (PMC7264146; doi:10.1038/s41422-020-0319-4)
Supplement: Supplementary file 5 — Supplementary Figure S5 [file 41422_2020_319_MOESM5_ESM.pdf]

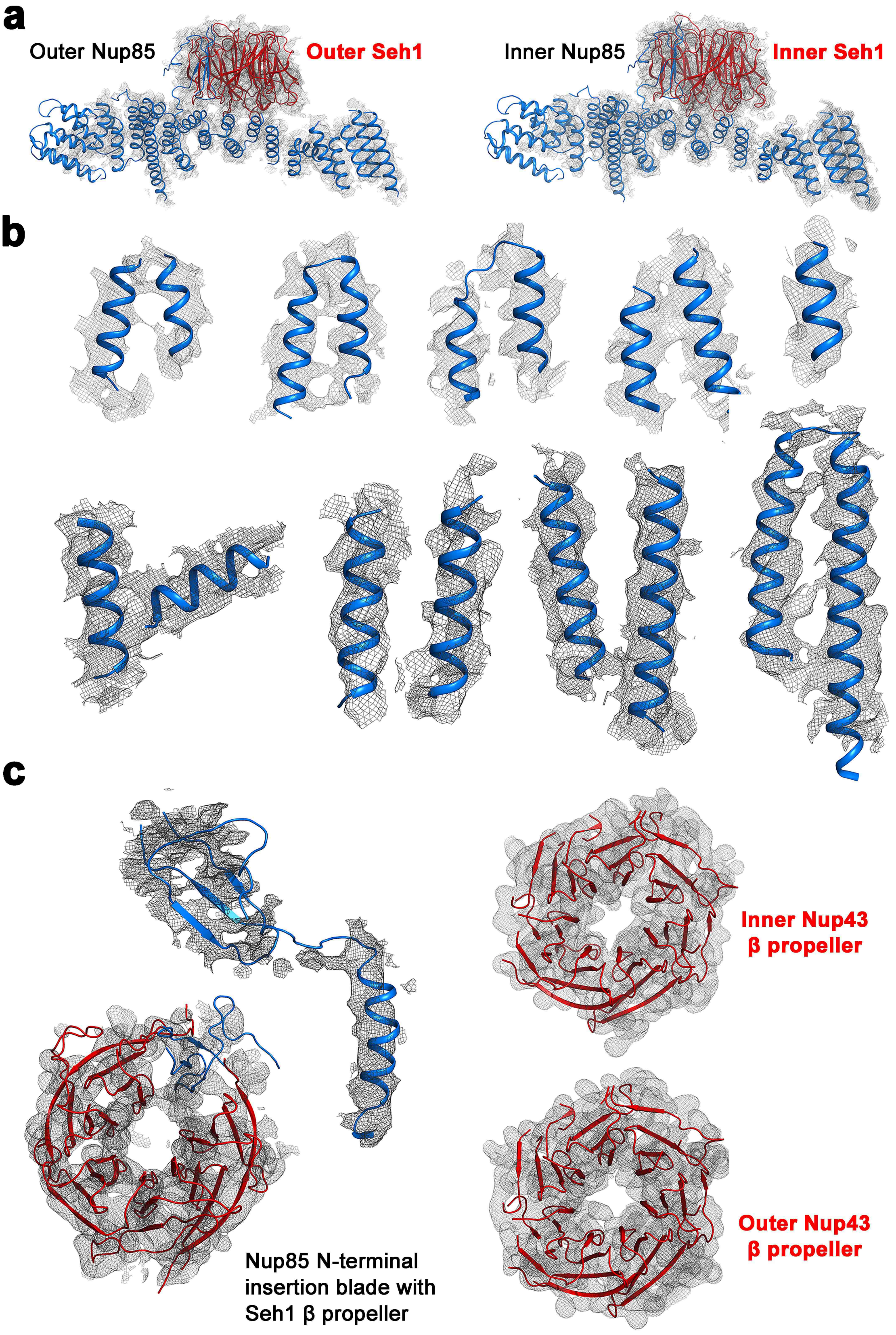

Supplementary information, Fig. S5 | Representative EM density maps for Nup85, Seh1 and Nup43 of the Core domain. **a**, The overall EM density map of Nup85 and

Seh1. The EM density maps for Nup85 and Seh1 from the inner and outer Y complexes are shown in the left and right panels, respectively. **b**, Representative EM density maps for a number of discrete α-helices from inner Nup85. The EM density maps for four pairs of HEAT repeats are shown in the upper panels. **c**, The EM density maps for the β-propeller domains of inner Seh1 (left panel) and inner and outer Nup43 (right panels). One blade in the Seh1 β-propeller comes from Nup85. All EM density maps in this figure were prepared using the masked Core region map with a contour level between 15σ and 25σ.
