# Supplementary material for: Structure of the cytoplasmic ring of the Xenopus laevis nuclear pore complex by cryo-electron microscopy single particle analysis
Source: Cell Res. 2020 May 6;30(6):520–31. doi: 10.1038/s41422-020-0319-4 (PMC7264146; doi:10.1038/s41422-020-0319-4)
Supplement: Supplementary file 6 — Supplementary Figure S6 [file 41422_2020_319_MOESM6_ESM.pdf]

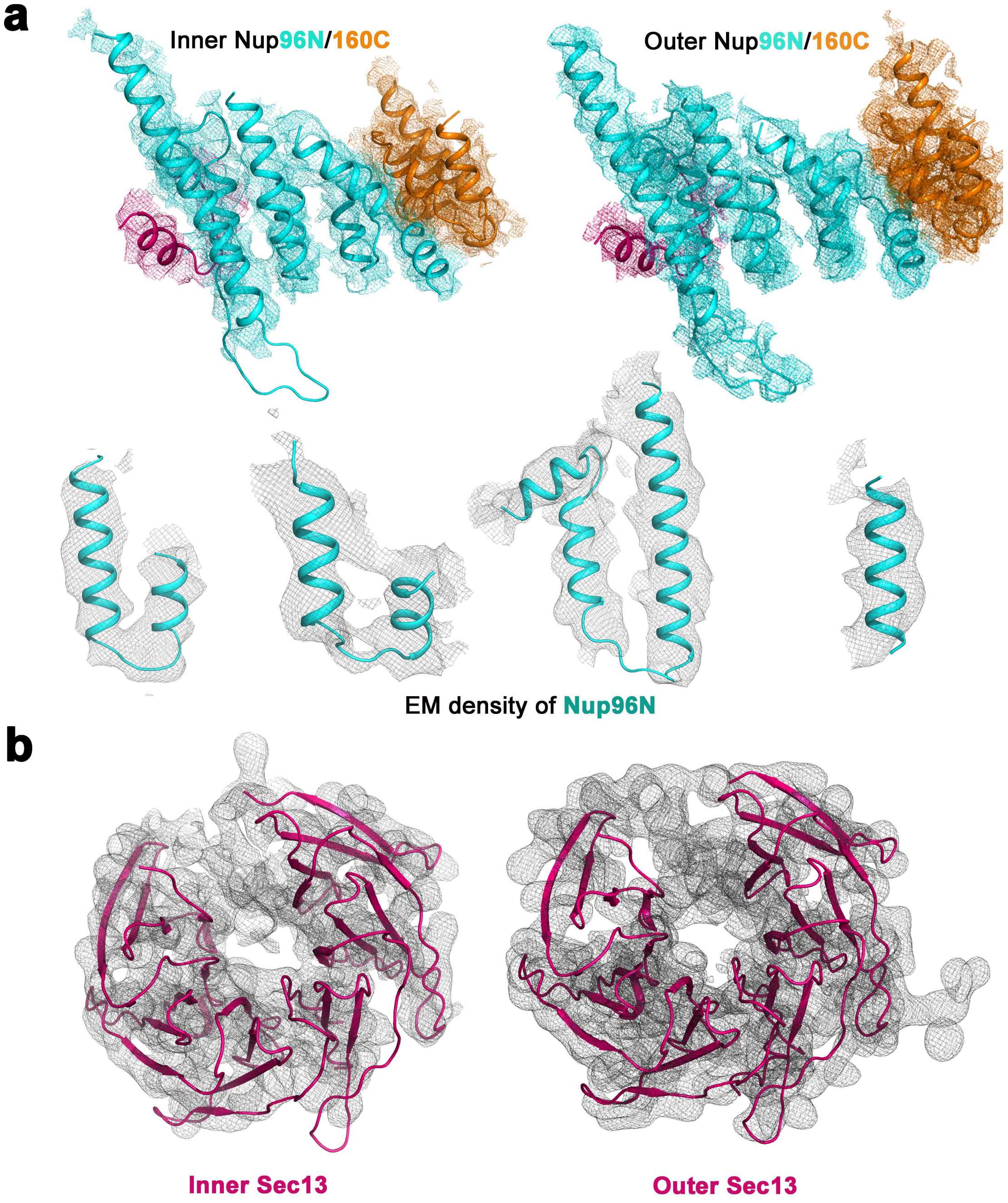

Supplementary information, Fig. S6 | Representative EM density maps for Nup96N/160C and Sec13 of the Core domain. **a**, The EM density map of Nup96N/160C. The EM density maps for the NTD of Nup96 and the CTD of Nup160 are shown in the upper panels. Representative EM density maps are shown in the lower panels for selected α-helices of the inner and outer Nup96N/160C. **b**, The EM density maps of the β-propeller domains in the inner (left panel) and outer (right panel) Sec13. Each β-propeller only has six blades. The unoccupied density for one blade in both

cases comes from Nup96. All EM density maps in this figure were prepared using the masked Core region map with a contour level between 15σ and 25σ.
