# Supplementary material for: Structure of the cytoplasmic ring of the Xenopus laevis nuclear pore complex by cryo-electron microscopy single particle analysis
Source: Cell Res. 2020 May 6;30(6):520–31. doi: 10.1038/s41422-020-0319-4 (PMC7264146; doi:10.1038/s41422-020-0319-4)
Supplement: Supplementary file 7 — Supplementary Figure S7 [file 41422_2020_319_MOESM7_ESM.pdf]

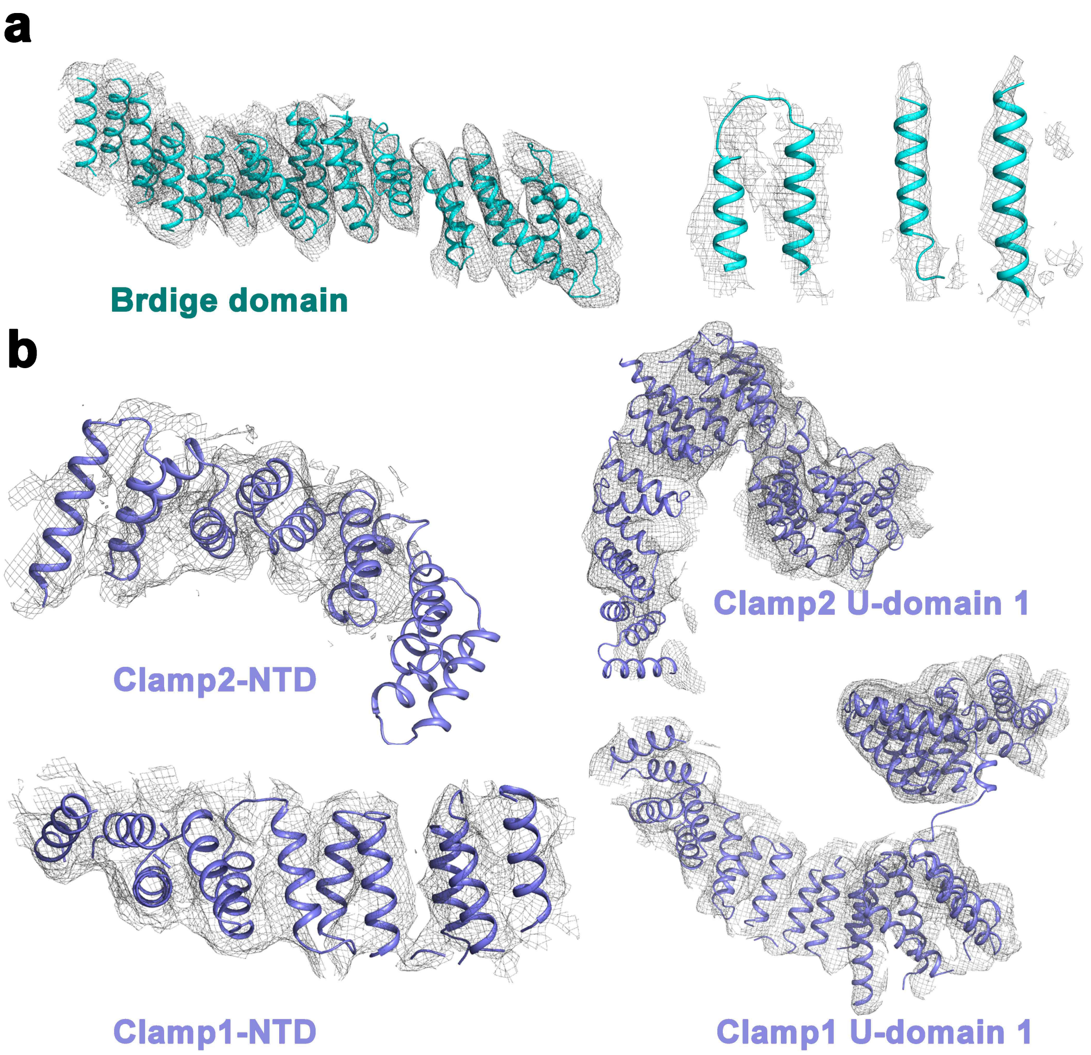

Supplementary information, Fig. S7 | The EM density maps for the Nup358 complex. **a**, The EM density maps for the bridge domain (left panel) and selected α-helices from the bridge domain (right panel). **b**, Representative EM density maps for selected regions of the Nup358 complex. Shown here are the EM maps for the N-terminal helices of Clamp-1 and Clamp-2 and the U-domain from Clamp-1. All EM density maps in this figure were prepared using the masked Nup358-containing region map with a contour level between 15σ and 25σ.
