# Supplementary material for: Structure of the cytoplasmic ring of the Xenopus laevis nuclear pore complex by cryo-electron microscopy single particle analysis
Source: Cell Res. 2020 May 6;30(6):520–31. doi: 10.1038/s41422-020-0319-4 (PMC7264146; doi:10.1038/s41422-020-0319-4)
Supplement: Supplementary file 8 — Supplementary Figure S8 [file 41422_2020_319_MOESM8_ESM.pdf]

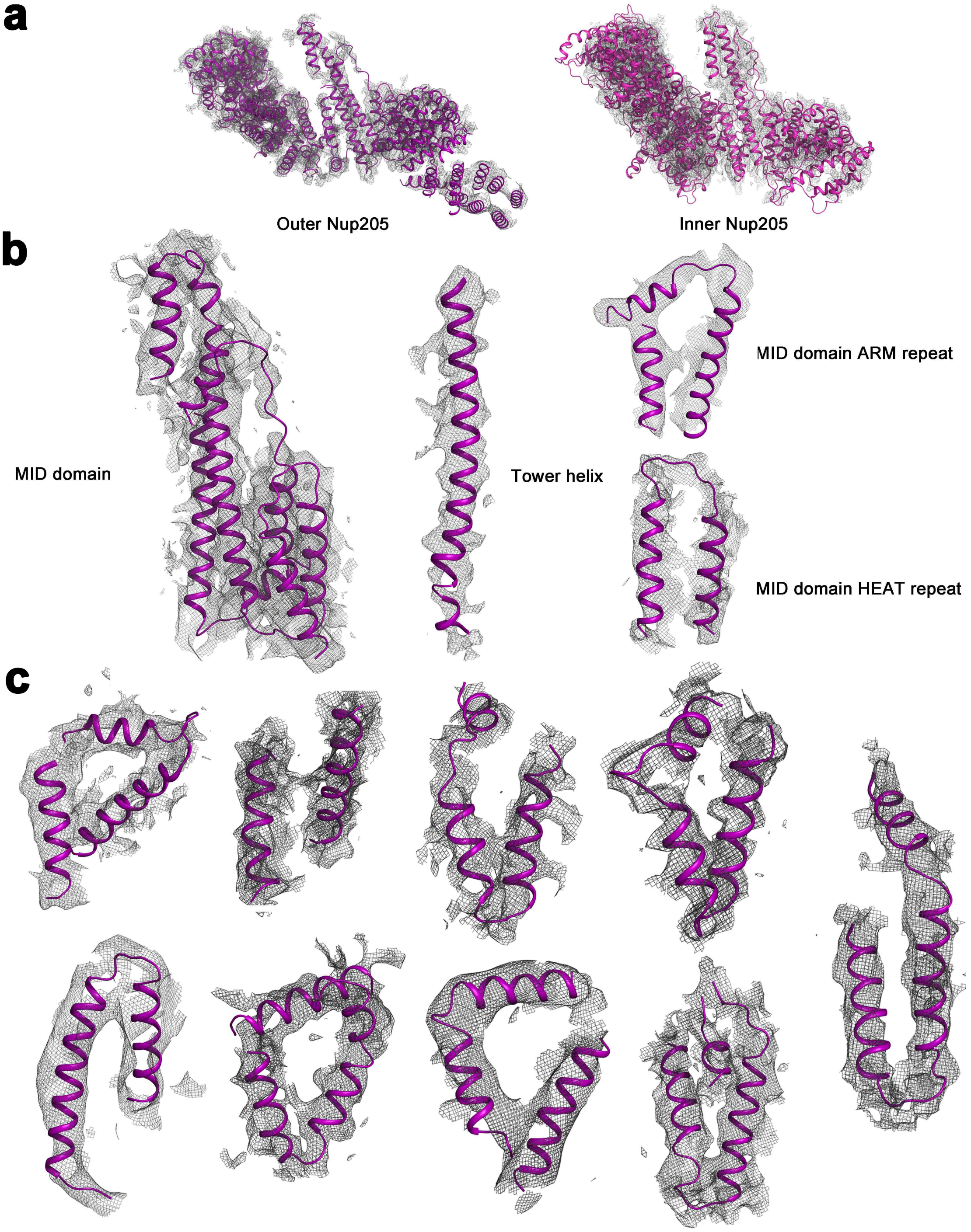


**Supplementary information, Fig. S8 | The EM density maps for Nup205.** **a**, The overall EM density maps for the outer and inner Nup205. **b**, The EM density maps for the MID domain of outer Nup205, which contains a characteristic Tower helix. The ARM repeat and HEAT repeat exhibit contrasting features for their EM density. **c**, Representative EM density maps for a number of discrete α-helices of outer Nup205.

All EM density maps in this figure were prepared using the masked Core region map with a contour level between 15σ and 25σ.
