# Supplementary material for: Structure of the cytoplasmic ring of the Xenopus laevis nuclear pore complex by cryo-electron microscopy single particle analysis
Source: Cell Res. 2020 May 6;30(6):520–31. doi: 10.1038/s41422-020-0319-4 (PMC7264146; doi:10.1038/s41422-020-0319-4)
Supplement: Supplementary file 9 — Supplementary Figure S9 [file 41422_2020_319_MOESM9_ESM.pdf]

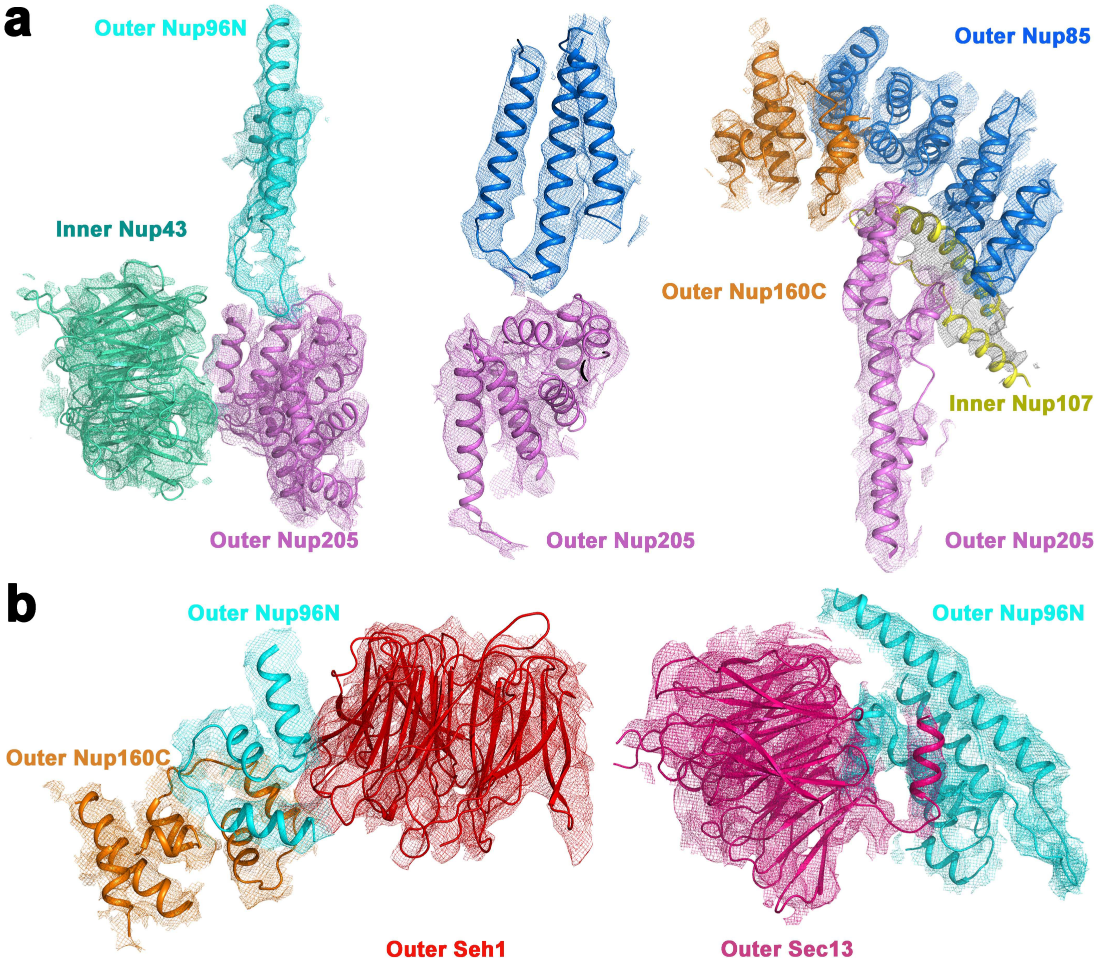

Supplementary information, Fig. S9 | The EM density maps for representative interfaces in the CR subunit. **a**, Representative EM density maps for proteins that interact with Nup205. Shown here are the local EM density maps for the proteins that interact with the NTD (left panel), the Tower helix (middle panel) and TAIL (right panel) of outer Nup205. The individual protein components and their associated density maps are color-coded. **b**, The EM density maps surrounding the N-terminal domain of outer Nup96. All EM density maps in this figure were prepared using the masked Core region map with a contour level between 15σ and 25σ.
