# Supplementary material for: Structure of the cytoplasmic ring of the Xenopus laevis nuclear pore complex by cryo-electron microscopy single particle analysis
Source: Cell Res. 2020 May 6;30(6):520–31. doi: 10.1038/s41422-020-0319-4 (PMC7264146; doi:10.1038/s41422-020-0319-4)
Supplement: Supplementary file 10 — Supplementary Figure S10 [file 41422_2020_319_MOESM10_ESM.pdf]

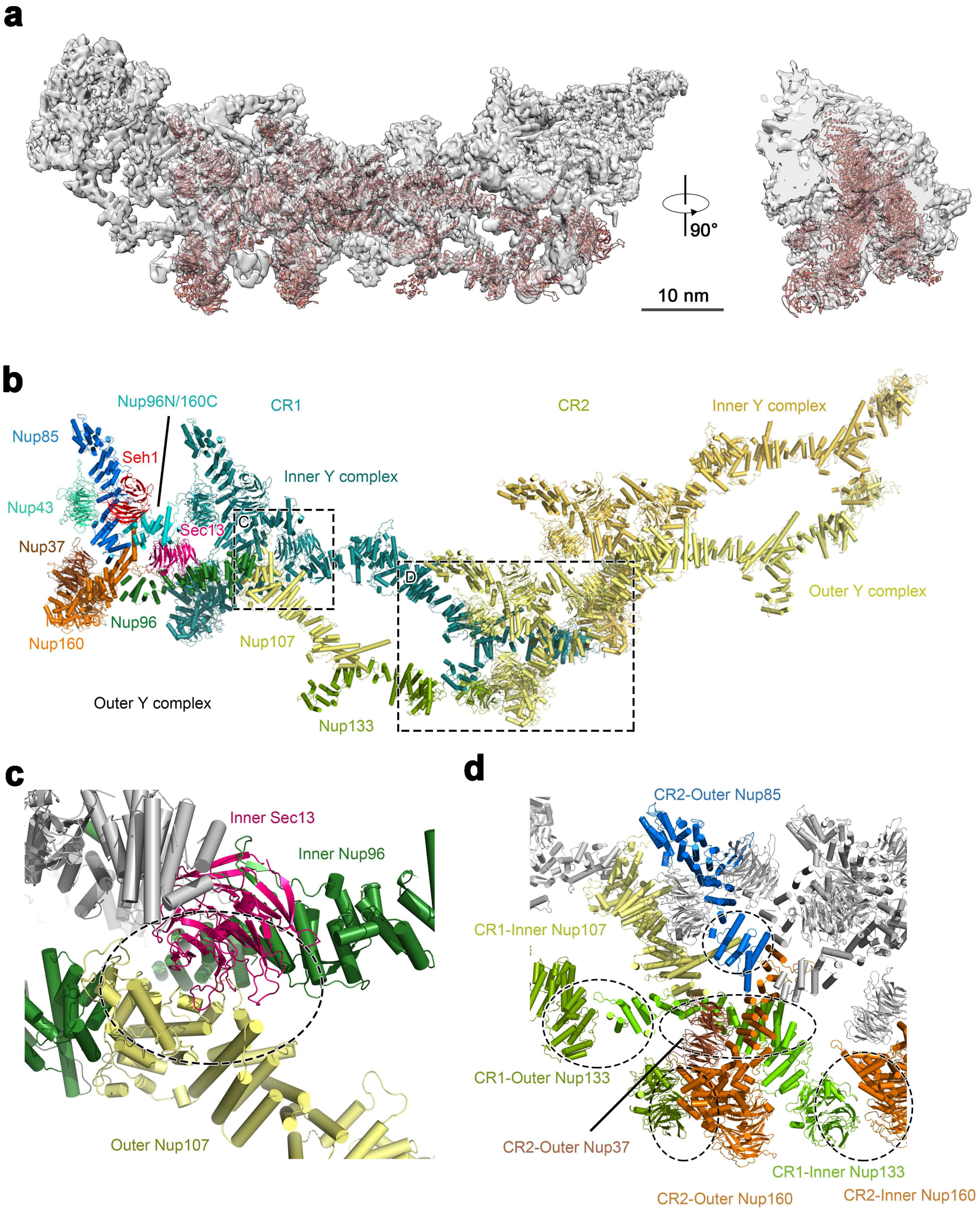

Supplementary information, Fig. S10 | Interactions among the Y complexes. **a**, The final coordinates of the inner and outer Y complexes are placed into the EM density map. Two views are shown. **b**, Interactions among the Y complexes within the same CR subunit and between two adjacent subunits. The inner and outer Y complexes interact with each other within the same subunit. A pair of Y complex from one subunit associate with its counterpart from the adjacent subunit in a head-to-tail fashion. **c**, A close-up view on the interface between the inner and outer Y complexes within the

same subunit. **d**, A close-up view on the interface between two neighboring CR subunits. Five areas of interactions are indicated by dashed oval circles.
