# Supplementary material for: Structure of the cytoplasmic ring of the Xenopus laevis nuclear pore complex by cryo-electron microscopy single particle analysis
Source: Cell Res. 2020 May 6;30(6):520–31. doi: 10.1038/s41422-020-0319-4 (PMC7264146; doi:10.1038/s41422-020-0319-4)
Supplement: Supplementary file 11 — Supplementary Figure S11 [file 41422_2020_319_MOESM11_ESM.pdf]

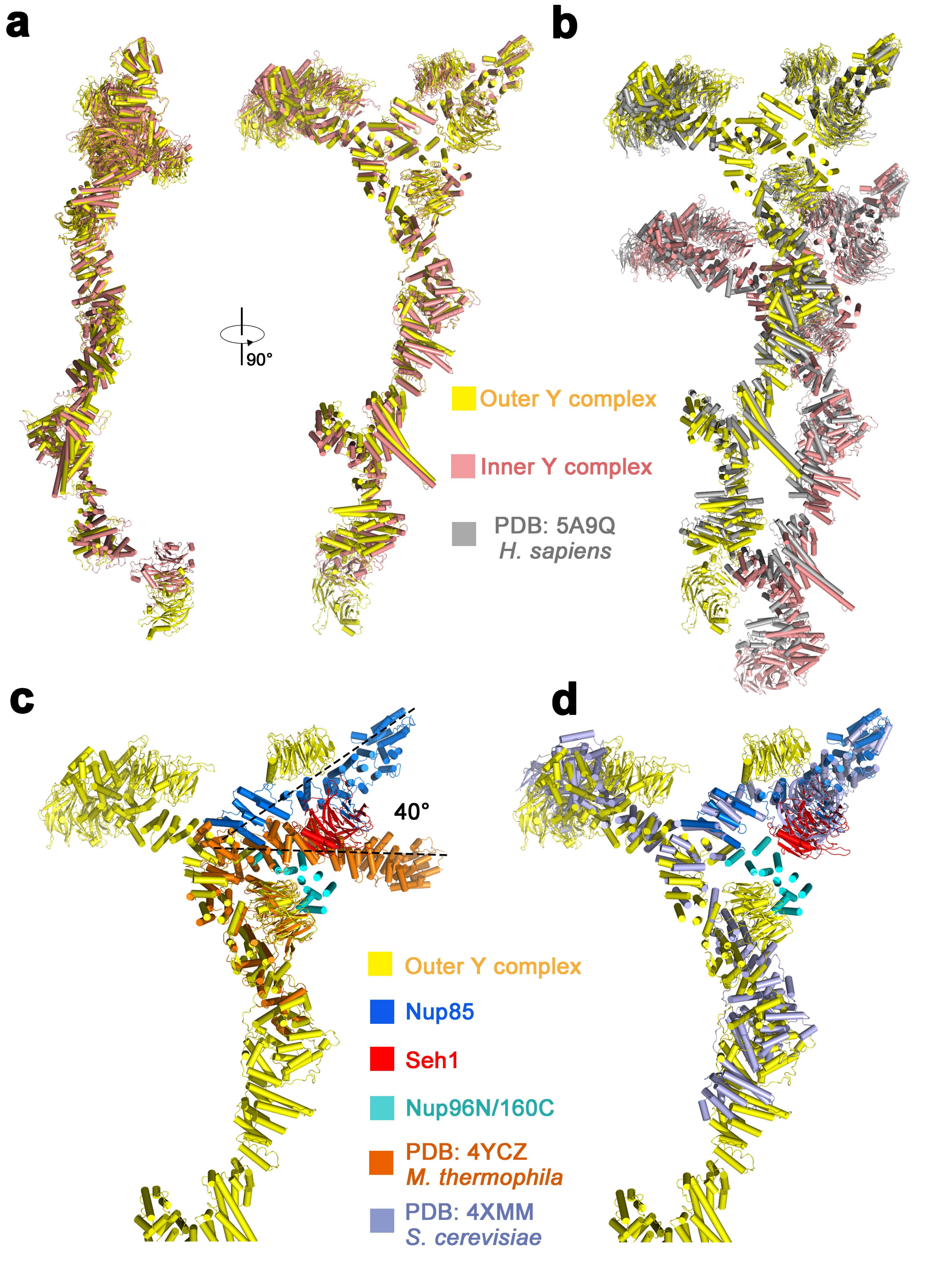

Supplementary information, Fig. S11 | Structural comparison among the Y complexes. **a**, Structural comparison between the inner and outer Y complexes within the same CR subunit. Two perpendicular views are shown. The inner and outer Y complexes are colored salmon and yellow, respectively. **b**, Structural comparison between the *X. laevis* Y complex and the *H. sapiens* Y complex. Coordinates of the

inner and outer Y complexes from *X. laevis* are superimposed with those from *H. sapiens*[1]. The overall conformation and main features of the Y complexes remain very similar between these two species. The only notable difference is that the top and bottom faces of the Nup43 β-propeller in the *X. laevis* Y complexes are opposite of those in the human Y complexes. **c**, Structural comparison between the *X. laevis* Y complex with the *Myceliophthora thermophila* (*M. thermophila*) Y complex[2]. **d**, Structural comparison between the *X. laevis* Y complex with the *Saccharomyces cerevisiae* (*S. cerevisiae*) Y complex[3]. The overall conformation of the *X. laevis* Y complex is similar to that of the *S. cerevisiae* Y complex, but numerous local structural variations are present. There are no *S. cerevisiae* homologues for the *X. laevis* Nup43 and Nup37.

References

1 von Appen, A. *et al*. In situ structural analysis of the human nuclear pore complex. *Nature* **526**, 140-143, doi:10.1038/nature15381 (2015).

2 Kelley, K., Knockenhauer, K. E., Kabachinski, G. & Schwartz, T. U. Atomic structure of the Y complex of the nuclear pore. *Nat Struct Mol Biol* **22**, 425-431, doi:10.1038/nsmb.2998 (2015).

3 Stuwe, T. *et al*. Architecture of the nuclear pore complex coat. *Science* **347**, 1148-1152 (2015).
