# Supplementary material for: Structure of the cytoplasmic ring of the Xenopus laevis nuclear pore complex by cryo-electron microscopy single particle analysis
Source: Cell Res. 2020 May 6;30(6):520–31. doi: 10.1038/s41422-020-0319-4 (PMC7264146; doi:10.1038/s41422-020-0319-4)
Supplement: Supplementary file 12 — Supplementary Figure S12 [file 41422_2020_319_MOESM12_ESM.pdf]

Supplementary information, Fig. S12

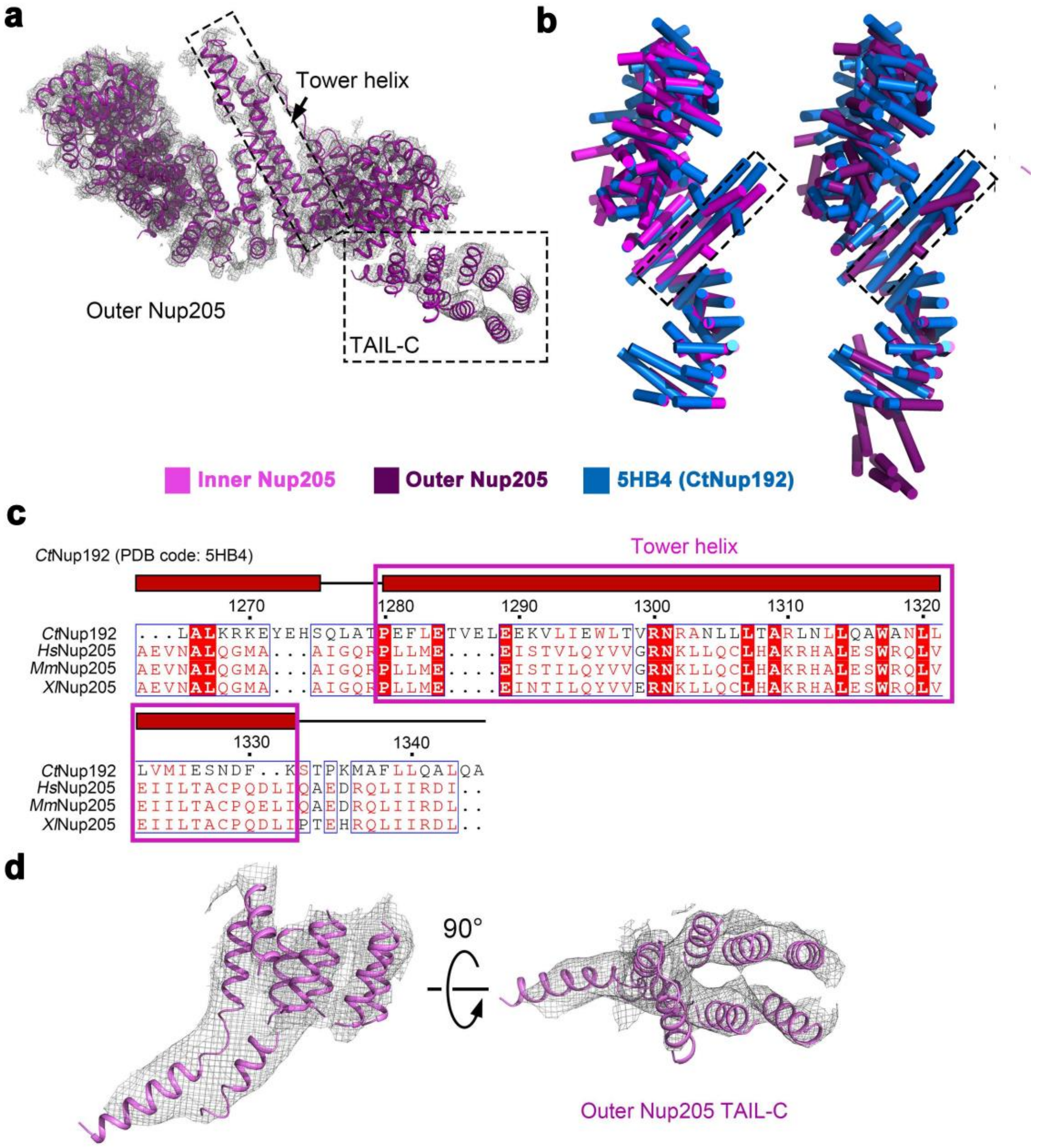

Supplementary information, Fig. S12 | The basis for the assignment of Nup205. **a**, The overall EM density map is more consistent with that for Nup205. The presence of continuous EM density for additional α-helices beyond the predicted C-terminal end of Nup188 favors the assignment of Nup205. Additionally, the length of the EM density for the Tower helix is consistent with that of CtNup192 or *X. laevis* Nup205. **b**, Comparison of the Tower helices between *X. laevis* Nup205 and CtNup192. Inner and outer Nup205 is compared to CtNup192 in the left and right panels, respectively. **c**, The length of the predicted Tower helix in Nup205 is similar to that of CtNup192 (PDB code: 5HB4)[1]. Shown here is a sequence alignment of the Tower helix region between CtNup192 and Nup205 of three vertebrate species[2,3]. **d**, The EM density

that is connected to the rest of the EM density for Nup205 or Nup188. Seven α-helices (two of them are longer than the other five and are slightly bent in the middle) can be accommodated by the EM density map. Based on the location and continuity of the EM density, these seven α-helices are assigned to the C-terminus of Nup205 (TAIL-C).

Reference

1 Lin, D. H. *et al.* Architecture of the symmetric core of the nuclear pore. *Science* **352**, aaf1015, doi:10.1126/science.aaf1015 (2016).

2 Madeira, F. *et al.* The EMBL-EBI search and sequence analysis tools APIs in 2019. *Nucleic Acids Res* **47**, W636-W641, doi:10.1093/nar/gkz268 (2019).

3 Robert, X. & Gouet, P. Deciphering key features in protein structures with the new ENDscript server. *Nucleic Acids Res* **42**, W320-324, doi:10.1093/nar/gku316 (2014).
