# Supplementary material for: Structure of the cytoplasmic ring of the Xenopus laevis nuclear pore complex by cryo-electron microscopy single particle analysis
Source: Cell Res. 2020 May 6;30(6):520–31. doi: 10.1038/s41422-020-0319-4 (PMC7264146; doi:10.1038/s41422-020-0319-4)
Supplement: Supplementary file 13 — Supplementary Table S1 [file 41422_2020_319_MOESM13_ESM.pdf]

Supplementary information, Table S1 | Statistics of cryo-EM data collection and analysis.

**Data collection**				
EM equipment	Titan Krios			
Voltage (kV)	300			
Detector	Gatan K2			
Energy filter	Gatan GIF Quantum, 20 eV slit			
Pixel size (Å)	2.222			
Magnification	22,502			
Data set	Tilt0	Tilt30	Tilt45	Tilt55
Exposure rate ($e^-/(s·Å^2)$)	2.612	2.612	2.612	2.612
Number of frames	40	46	56	70
Total Electron exposure ($e^-/Å^2$)	52	60	78	91
Defocus range (μm)	-1.0 ~ -4.0			
Total Number of images	4,606	4,186	4,158	10,111
Selected Number of images	3,250	2,888	2,745	3,516
Software	AutoEMation2			
**Reconstruction**				
Software	RELION2.1/RELION3.0-beta/THUNDER			
Number of used Particles	133,125	198,099	142,938	142,385
Symmetry/Final resolution (Å)	C1/8.0			

Masked regions	Core region	Nup358 region	Nup214 region
Number of Micrographs used	12,399	12,399	12,399
Number of extracted particles	1,721,077	1,721,077	1,721,077
Final number of particles	616,547	387,233	552,066
Unmasked Resolution (Å) (0.5/0.143)	8.0/6.6	9.2/7.9	9.3/7.9
Masked Resolution (Å) (0.5/0.143)	7.3/5.4	8.4/6.7	8.8/7.6
Local Resolution Ranges (Å)	50-4.5	50-5.0	50-6.1
Resolution Range due to anisotropy (Å)	6.2-5.0	8.3-6.2	8.8-7.1
Final Resolution (Å)	5.5	7.1	7.9
Map sharpening B-factor ($Å^2$)	-200	-400	-400
Accuracy of rotation (˚)	2.23	2.55	5.02
Accuracy of translation (pixels)	0.99	1.39	1.90
EMDB number	emd-0909	emd-0910	emd-0911
**Model building and refinement**			
Model building software	Coot		
Refinement software	PHENIX		
Protein residues	16,161		
Side chains	N/A		
**Validation**			
CaBLAM outliers	1.97%		

PDB accession code		6LK8	
R.m.s deviations			
Bonds length (Å)	0.008	0.010	0.006
Bonds Angle (˚)	1.23	1.21	1.04
Ramachandran plot statistics (%)			
Preferred	88.66	85.33	87.70
Allowed	9.68	13.80	10.57
Outlier	1.66	0.87	1.73
