# Supplementary material for: Structure of the cytoplasmic ring of the Xenopus laevis nuclear pore complex by cryo-electron microscopy single particle analysis
Source: Cell Res. 2020 May 6;30(6):520–31. doi: 10.1038/s41422-020-0319-4 (PMC7264146; doi:10.1038/s41422-020-0319-4)
Supplement: Supplementary file 14 — Supplementary Table S2 [file 41422_2020_319_MOESM14_ESM.pdf]

Supplementary information, Table S2 | Summary of model building for the CR subunit of *X. laevis* NPC.

	Molecule **Vertebrates**/Yeasts	Copy No.		Length *X. laevis*	UniProt No. *X. laevis*	PDB code	Modeling	Model length	Resolution (Å)	Chain ID Outer/Inner
**Y complex**	**Nup85**/Nup85	2		653	Q68FJ0	3F3F, 4XMM	HM	~600	4.5~6.0	B/b
	**Nup160**/Nup120	2		1435	A0A1L8GIX3	4GQ2, 4XMM	HM	~1100	5.0~10.0	E/e
	**Nup96**/Nup145C	2		923	A0A1L8HBE3	3IKO, 4XMM	HM	~510	4.5~6.0	G/g
			**Nup96N/160C**	-	-	-	PA	~190	4.5~6.0	L/l
	**Nup107**/Nup84	2		916	A2RV69	3IKO, 3I4R	HM	~770	5.0~7.0	I/i
	**Nup133**/Nup133	2		1140	A0A1L8H1I9	1XKS, 3I4R	HM	~1000	6.5~9.0	J/j
	**Sec13**/Sec13	2		320	Q7ZYJ8	3BG1	HM	~280	4.5~6.0	H/h
	**Seh1**/Seh1	2		360	Q4FZW5/Q6GNF1	3F3F	HM	~310	4.5~6.0	D/d
	**Nup43**/-	2		375	Q05AW3	4I79	HM	~370	4.5~6.0	C/c
	**Nup37**/-	2		326	Q66IZ6	4GQ2	HM	~320	6.5~9.0	F/f
**Nup358**	**Nup358**/ -	4?		2905	A0A1L8HGL2			~810	6.0~12.5	
			**Bridge domain**			-	PA	~410	6.0~8.5	Q
			**Clamp-1**			4GA0	HM	~200	6.0~8.5	S
			**Clamp-2**			4GA0	HM	~200	7.0~10.5	U
**Nup205**	**Nup205**/Nup192	2		2011	Q642R6	5HB4	HM	~1770	4.5~6.0	A/a
			**TAIL-C**				PA	~150	4.5~6.0	A/a
**Nup214 complex**	**Nup214**/Nup159	2?		2037	Q9PVZ2	5C3L	PA	~90	7.5~10.5	N
	**Nup88**/Nup82	2?		728	Q4KLQ6	5C3L, 5CWW	PA	~450		M, R
	**Nup62**/Nsp1	2?		547	Q91349	5C3L	PA	~90		O
			**CCS2**			-	PA	~90	7.5~10.5	X, Y, Z

Under the column labeled "Molecule", names of the proteins from vertebrates and yeasts are shown, with vertebrate components in bold. HM, homology modelling; PA, poly-alanine model. Nup96N/160C, the bridge domain of the Nup358 complex, TAIL-C of Nup205 and CCS2 of the Nup214 complex were *ab initial* modeled. The remaining components of Nup214 in the grey region were not docked in the EM density map.
